# Prevalence and Characteristics of CKD in the US Military Health System: A Retrospective Cohort Study

**DOI:** 10.1016/j.xkme.2022.100487

**Published:** 2022-05-23

**Authors:** James D. Oliver, Robert Nee, Lindsay R. Grunwald, Amanda Banaag, Meda E. Pavkov, Nilka Ríos Burrows, Tracey Pérez Koehlmoos, Eric S. Marks

**Affiliations:** 1 Nephrology Service, Department of Medicine, Walter Reed National Military Medical Center, Bethesda, MD; 2 Henry M. Jackson Foundation, Bethesda, MD; 3 Centers for Disease Control and Prevention, Atlanta, GA; 4 Department of Preventive Medicine and Biostatistics, Uniformed Services University of the Health Sciences, Bethesda, MD; 5 Department of Medicine, Uniformed Services University of the Health Sciences, Bethesda, MD

**Keywords:** Chronic kidney disease, ICD-9 codes, kidney disease epidemiology, Military Health System, military medicine

## Abstract

**Rationale & Objective:**

The US Military Health System (MHS) is a global health care network with a diverse population that is more representative of the US population than other study cohorts and with fewer disparities in health care access. We aimed to examine the prevalence of chronic kidney disease (CKD) in the MHS and within demographic subpopulations.

**Study Design:**

Multiple cross-sectional analyses of demographic and claims-based data extracted from the MHS Data Repository, 1 for each fiscal year from 2006-2015.

**Setting & Population:**

Multicenter health care network including active-duty military, retirees, and dependents. The average yearly sample size was 3,285,348 individuals.

**Exposures:**

Age, sex, race, active-duty status, and active-duty rank (a surrogate for socioeconomic status).

**Outcome:**

CKD, defined as the presence of matching *International Classification of Diseases, Ninth Revision*, codes on either 1 or more inpatient or 2 or more outpatient encounters.

**Analytical Approach:**

*t* test for continuous variables and χ^2^ test for categorical variables; multivariable logistic regression for odds ratios.

**Results:**

For 2015, the mean (standard deviation) age was 38 (16). Crude CKD prevalence was 2.9%. Age-adjusted prevalence was 4.9% overall—1.9% active-duty and 5.4% non–active-duty individuals. ORs for CKD were calculated with multiple imputations to account for missing data on race. After adjustment, the ORs for CKD (all *P* < 0.001) were 1.63 (95% CI, 1.62-1.64) for an age greater than 40 years, 1.16 (95% CI, 1.15-1.17) for Black race, 1.15 (95% CI, 1.14-1.16) for senior enlisted rank, 0.94 (95% CI, 0.93-0.95) for women, and 0.50 (95% CI, 0.49-0.51) for active-duty status.

**Limitations:**

Retrospective study based on *International Classification of Diseases, Ninth Revision*, coding.

**Conclusions:**

Within the MHS, older age, Black race, and senior enlisted rank were associated with a higher risk of CKD, whereas female sex and active-duty status were associated with a lower risk.

## Graphical abstract

**Figure:**
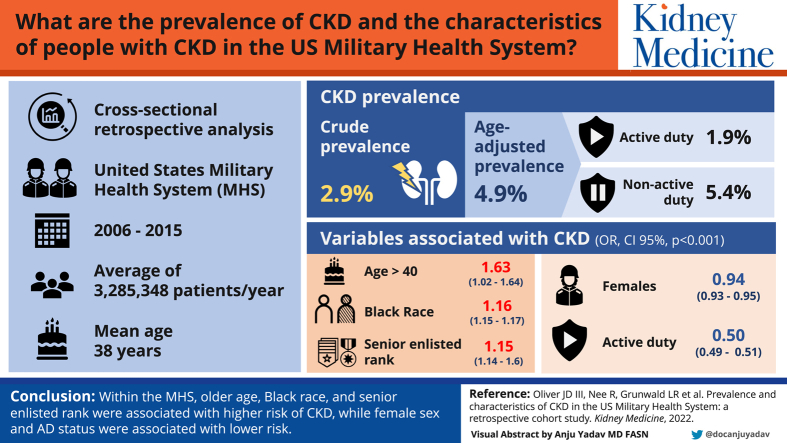

**Plain-Language Summary**

Studies of databases from health care systems provide insight on the impact of chronic kidney disease (CKD) on various populations. We examined CKD in the US Department of Defense Military Health System (MHS), which provides universal coverage to a large, diverse population with characteristics similar to that of the general US population. On examining the records of more than 3.3 million individuals in the MHS, we found that 2.9% had a diagnosis of CKD. It was more likely to occur in older people and those of Black race, and it was less likely to occur in women and those who were in the active-duty military. Further study will provide information on the quality of CKD health care and outcomes in the MHS.

Chronic kidney disease (CKD) is a common condition that presents a serious and growing challenge to population health with increased risks for multiple adverse outcomes, including kidney failure, cardiovascular disease, hospitalizations, and death.^1^ In the United States, kidney disease is the ninth leading cause of death,^2^ with an adjusted mortality that is almost 3 times that of individuals without CKD. An analysis of the 2016 Global Burden of Disease study in the United States revealed that the burden of CKD has increased from 2002-2016 at a faster pace than other noncommunicable diseases.^3^ Using data from the National Health and Nutrition Examination Survey, Coresh et al^4^ reported that the prevalence of CKD in the United States increased from 10% to 13% between 1988-1994 and 1999-2004. A disproportionate amount of this increase is attributed to diabetes and hypertension, which, in turn, is a consequence of higher exposure to metabolic and dietary risk factors, population growth, and aging. Expenditures on CKD constitute 23% of the Medicare budget.^5^ The impact of CKD on non-Medicare populations younger than 65 years in the United States is not well understood and depends largely on extrapolations from surveys such as the National Health and Nutrition Examination Survey or from large databases (eg, Veterans Affairs Health System and Optum Clinformatics).

The Military Health System (MHS) is a global, comprehensive, integrated medical network within the US Department of Defense serving 9.6 million beneficiaries, including active-duty service members, retirees, and family members, with an annual budget of US $53 billion.^6^ The MHS delivers care through both a direct-care/health maintenance organization system provided at the Department of Defense military treatment facilities and a purchased-care/preferred provider organization system provided at civilian facilities. The vast majority of kidney failure–related care (dialysis and transplant) is provided outside of the MHS through Medicare. Although often confused with the Department of Veterans Affairs (VA) Health System, the MHS is a distinct entity with separate facilities, and MHS beneficiaries more closely mirror the general US population with regard to age, sex, and socioeconomic characteristics.^7^

The MHS provides universal coverage for its beneficiaries under a program called TRICARE; it has been cited as a model of equitable health care access across socioeconomic and racial groups.^8^ Multiple studies have demonstrated mitigation of racial disparities in the MHS.^9^ As such, the MHS is an important and untapped source of cross-sectional and longitudinal information on CKD and other chronic illnesses. In this report, we present a comprehensive description of the prevalence and demographics of CKD in the MHS.

## Methods

### Study Population

This was a retrospective cross-sectional study. We extracted deidentified patient data for fiscal years (FYs) 2006-2015 from the MHS Data Repository, the administrative claims database for all care received through the MHS. Each FY (October 1-September 30) is based on the budget calendar of the US Federal Government. Each FY was analyzed separately. The MHS Data Repository does not capture health care delivery in combat zones or care received in the VA system. All individuals were in the TRICARE Prime managed care option.

Individuals older than 18 years were classified as either active-duty or non–active-duty (ie, dependent or retiree) on the basis of their designation for the FY in the database. Before entering active-duty status, individuals undergo a screening process, which includes self-reported health history, blood pressure, physical examination, and dipstick urinalysis. Serum creatinine and quantitative albuminuria are not routinely tested as part of the screening. Therefore, active-duty individuals represent a distinct cohort within the MHS for assessing chronic illness.

The Uniformed Services University institutional review board deemed this study exempt. Informed consent was not required because of the use of deidentified data.

### CKD

The prevalence of CKD was the primary outcome of interest. CKD diagnosis was based on encounters with 1 or more matching *International Classification of Diseases, Ninth Revision*, (ICD-9) codes for CKD. The full list of codes used is provided in Table S1. CKD diagnosis included kidney failure, which was defined as having an ICD-9 or Current Procedural Terminology code for kidney failure, chronic hemodialysis, chronic peritoneal dialysis, or kidney transplant (2015 was the last year the MHS used ICD-9 before converting to *International Classification of Diseases, Tenth Revision*). For the calculation of CKD prevalence, we used the method of Hebert et al,^10^ which was also used by the US Renal Data System. The numerator was the number of individuals with at least 1 inpatient or 2 outpatient CKD codes. The denominator was the number of individuals with at least 1 inpatient or 2 outpatient encounters. A sensitivity analysis was performed using alternative definitions for the numerator (number of individuals with at least 1 CKD code) and denominator (number of individuals with at least 1 encounter). The assignment of comorbid conditions was also done on the basis of 1 inpatient or 2 outpatient ICD-9 codes during the same FY. Age-adjusted prevalence was calculated using data from the 2015 US Census Bureau American Community Survey.^11^

### Rank

The rank of either the patient (for active-duty and retirees) or the patient’s military sponsor (for dependents) was used as a surrogate for socioeconomic status.^12^ Rank categories were defined as junior enlisted (grade, E1-E4; 2015 base salary range, US $18,564-$29,424), senior enlisted (grade, E5-E9; 2015 base salary range, US $26,436-$91,020), junior officer (grades, O1-O3 and W1-W2; 2015 base salary range, US $34,416-$76,380), and senior officer (grades, O4 and above and W3 and above; 2015 base salary range, US $39,216-$237,156).

### Race

The MHS Data Repository categorizes race into 6 classifications: White, Asian or Pacific Islander, Black, American Indian or Alaska Native, Other, and Unknown. A significant portion of data on race for non–active-duty individuals is not recorded, and is, therefore, coded as “Missing.” Data on ethnicity in the MHS Data Repository are sparse and were not considered for analysis.

### Statistical Analysis

Descriptive statistics were calculated as mean (standard deviation) or median (interquartile range) for continuous variables and proportions for categorical variables. Statistical comparisons were made using *t* test for continuous variables and χ^2^ test for categorical variables. We calculated odds ratios for having CKD using multivariable logistic regression, adjusted for age, sex, race, active-duty versus non–active-duty status, and rank. As a sensitivity analysis, we repeated the logistic regression using imputed data on race as described below. Because of the large data sets, a more stringent definition of *P* < 0.001 was used for statistical significance. Analyses were performed using SAS version 9.4 (SAS Institute Inc).

To account for missing data on race, we calculated odds ratios in 2 ways: first, by excluding individuals with missing values (ie, complete case analysis) and, second, by imputing the missing race using the method of multiple imputation by fully conditional specification.^13^ We imputed race in SAS using procedure PROC MI. The results from each imputation were pooled to generate a single set of estimated parameters, which were then exponentiated to determine the odds ratios. The use of multiple imputation in our analyses was based on the assumption that the missing data on race were missing at random given that the reason for missing data was likely explained by other observed characteristics, such as sex and active-duty versus non–active-duty status.^14^

## Results

We identified 7,447,373 unique individuals from FY 2006–FY 2015. The average yearly cohort size was 3,285,348. Of these, 762,000 (10.2%) individuals had encounters in all 10 years and 3,014,738 (40.4%) had encounters in 5 or more years. This report gives detailed analysis for the FY 2015 only; analyses of the other years yielded similar results and are shown in Tables S2-S5.

### FY 2015 Demographics

A cohort of 3,344,420 individuals (mean [standard deviation] age, 38 [16] years) were examined for the FY 2015 (Table 1). In the cohort, 44.7% individuals were women, 46.4% individuals were White, 12.7% individuals were Black, 4.0% individuals were Asian/Pacific Islander, 0.8% individuals were American Indian/Alaska Native, 2.5% individuals belonged to the Other category, and 5.7% individuals belonged to the Unknown category. Active-duty individuals comprised 43.2% of the cohort, with a majority (79.1%) being enlisted or dependents of enlisted.

**Table 1:** Demographic Characteristics of the Active-Duty Versus Non–Active-Duty Populations and of the Population With CKD Versus the Population with Non-CKD

	FY 2015 Total Population	Active-Duty	Non–Active-Duty	CKD	Non-CKD
No. (%)	3,344,420 (100.0%)	1,443,268 (43.2%)	1,901,152 (56.8%)	96,006 (2.9%)	3,248,414 (97.1%)
Age, mean (SD), y	37.6 (15.6)	28.3 (8.4)	44.7 (16.0)	52.9 (17.6)	37.2 (15.3)
Age, median (IQR), y	34 (24-50)	26 (22-34)	47 (31-57)	55 (41-63)	34 (24-49)
Female, n (%)	1,495,035 (44.7%)	249,445 (17.3%)	1,245,590 (65.5%)	51,096 (53.2%)	1,443,939 (44.5%)
Race, n (%)					
White	1,550,283 (46.4%)	1,023,370 (70.9%)	526,913 (27.7%)	27,659 (28.8%)	1,522,624 (46.9%)
Black	425,029 (12.7%)	249,026 (17.3%)	176,003 (9.3%)	14,356 (15%)	410,673 (12.6%)
Asian American/Pacific Islander	132,568 (4.0%)	79,211 (5.5%)	53,357 (2.8%)	2,746 (2.9%)	129,822 (4%)
American Indian/Alaska Native	25,374 (0.8%)	18,279 (1.3%)	7,095 (0.4%)	436 (0.5%)	24,938 (0.8%)
Other	83,804 (2.5%)	52,019 (3.6%)	31,785 (1.7%)	1,716 (1.8%)	82,088 (2.5%)
Unknown	191,065 (5.7%)	19,624 (1.4%)	171,441 (9.0%)	13,398 (14.0%)	177,667 (5.5%)
Missing	936,297 (28.0%)	1,739 (0.1%)	934,558 (49.2%)	35,695 (37.2%)	900,602 (27.7%)
Active-duty, n (%)	1,443,268 (43.2%)	1,443,268 (100.0%)	0 (0.0%)	9,635 (10.0%)	1,433,633 (44.1%)
Rank, n (%)					
Junior enlisted	804,234 (24.1%)	650,885 (45.1%)	153,349 (8.1%)	7,153 (7.5%)	797,081 (24.5%)
Senior enlisted	1,839,148 (55.0%)	526,308 (36.5%)	1,312,840 (69.1%)	71,245 (74.2%)	1,767,903 (54.4%)
Junior officer	363,963 (10.9%)	181,106 (12.6%)	182,857 (9.6%)	7,092 (7.4%)	356,871 (11%)
Senior officer	321,619 (9.6%)	69,642 (4.8%)	251,977 (13.3%)	10,469 (10.9%)	311,150 (9.6%)
Unknown/missing	15,456 (0.5%)	15,327 (1.1%)	129 (0.0%)	47 (0.0%)	15,409 (0.5%)
Crude CKD prevalence, n (%)	96,006 (2.9%)	9,635 (0.7%)	86,371 (4.5%)	96,006 (100%)	0 (0.0%)
Age-adjusted CKD prevalence, %	4.9%	1.9%	5.4%	NA	NA

Abbreviations: CKD, Chronic kidney disease; FY, fiscal year; IQR, interquartile range; NA, not applicable; SD, standard deviation.

A significant proportion of the cohort had missing data on race (n = 936,294; 28%); these were almost exclusively in the non–active-duty population (49.2% missing race in non–active-duty vs 0.1% missing in active-duty). In addition, those with missing race were older (40 vs 38 years) and more likely to be female (91.0% vs 44.7%) compared with the overall cohort (*P* < 0.001).

The active-duty population was younger (28 vs 45 years for active-duty vs non–active-duty). Nearly two-thirds (62.5%) of the active-duty population were between the ages of 18-29 years, compared with just 23.1% of the non–active-duty group (see Fig S1). Active-duty individuals were less likely to be female (17.3% vs 65.5%) and more likely to be White (70.9% vs 27.7%) than the non–active-duty individuals. The non-White, non–active-duty percentage was 45.6% after excluding missing race. A plurality of the active-duty population was in the junior enlisted rank (45.1%), whereas in the non–active-duty population of retirees and dependents, 69.1% of individuals were senior enlisted.

CKD individuals were older (53 vs 37 years), more likely to be female (53.2% vs 44.5%), more likely to be Black (15.0% vs 12.7%), less likely to be active-duty (10.0% vs 44.1%), and more likely to belong to the senior enlisted (74.2% vs 54.4%) or senior officer rank group (10.9% vs 9.6%) than their non-CKD counterparts (*P* < 0.001 for all). Within race groups, the plurality of CKD individuals (37.2%) was in the Missing group.

### CKD Prevalence

The unadjusted prevalence of CKD for FY 2015 based on ICD-9 codes was 2.9%, with an age-adjusted prevalence of 4.9% (Table 1). As a sensitivity analysis, we calculated additional estimates for crude prevalence using alternative definitions for the denominator and numerator: prevalence was 2.4% when using an alternative denominator (number of individuals with 1 or more encounter of any kind), 4.0% when using an alternative numerator (number of individuals with 1 or more CKD code of any kind), and 3.8% when using both the alternative numerator and denominator. As expected, CKD prevalence was significantly lower in the active-duty population than in the non–active-duty population, both unadjusted (0.7% vs 4.5%, *P* < 0.001) and age-adjusted (1.9% and 5.4%, *P* < 0.001).

**Figure 1:**
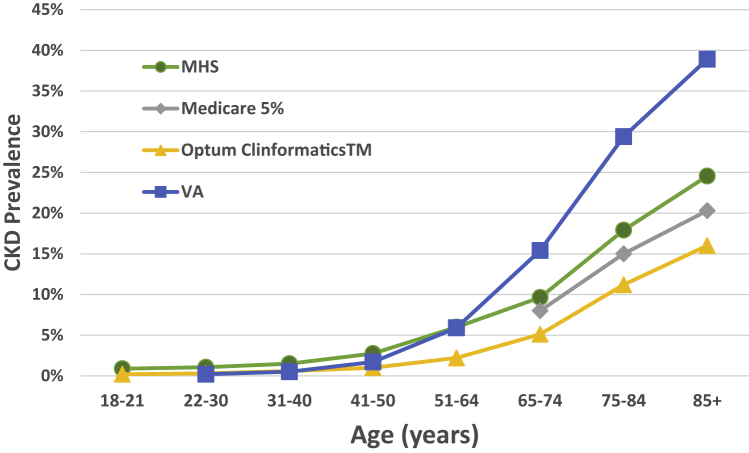
Chronic kidney disease prevalence versus age. Age-stratified chronic kidney disease prevalence (CKD) in the US Military Health System (MHS) versus Medicare 5%, Optum Clinformatics, and Department of Veterans Affairs (VA) (data from US Renal Data System 2017 Annual Data Report).^15^

Age-stratified CKD prevalence increased in monotonic fashion: from ages 18-21 years, it was 0.9%; from ages 22-30 years, it was 1.1%; from ages 31-40 years, it was 1.5%; from ages 41-50 years, it was 2.7%; from ages 51-64 years, it was 6.0%; from ages 65-74 years, it was 9.6%, from ages 75-84 years, it was 17.9%; and over the age of 84 years, it was 24.6%. The values are comparable to 2015 data from other large databases (Fig 1).^15^

The crude CKD prevalence for various subpopulations of active-duty status, sex, race, and rank are included in Tables S2-S5 and are summarized here. Unadjusted CKD prevalence was higher in women than men (3.2% vs 2.2%, *P* < 0.001), although the difference narrowed after age adjustment (4.7% vs 4.5%, *P* < 0.001). CKD prevalence was higher in women than in men in the active-duty population (1.2% vs 0.5%, *P* < 0.001) but lower in the non–active-duty population (3.6% vs 5.2%, *P* < 0.001). Among racial groups, individuals with Unknown (6.3%) and Missing (3.5%) race categories had the highest CKD prevalence, followed by Black race (3.0%). White, Asian-American/Pacific Islander, American Indian/Alaska Native, and Other races all had CKD prevalence from 1.6%-1.9%. CKD was more common in the older senior enlisted (3.5%) and senior officer (3.0%) ranks than in the junior enlisted (0.8%) and junior officer (1.8%).

### CKD in Active-Duty Population

As shown in Table 2, active-duty individuals with CKD were older than active-duty individuals without CKD (34 vs 28 years), although this age difference (5.5 years) was smaller than that seen between CKD versus non-CKD in the entire cohort (15.7 years, Table 1). Active-duty individuals with CKD were more likely to be women (31.4% vs 17.2%), more likely to be Black (27.3% vs 17.2%), and more likely to belong to senior rank groups than active-duty individuals without CKD.

**Table 2:** Demographics of CKD Versus Non-CKD in the Active-Duty Population

	FY 2015 Active-Duty	Active-Duty + CKD	Active-Duty + Non-CKD
No. (%)	1,443,268 (100%)	9,635 (0.7%)	1,433,633 (99.3%)
Age, mean (SD), y	28.3 (8.4)	33.8 (10.0)	28.3 (8.4)
Age, median (IQR), y	26 (22-34)	33 (25-41)	26 (22-34)
Female, n (%)	249,445 (17.3%)	3,021 (31.4%)	246,424 (17.2%)
Race, n (%)			
White	1,023,370 (70.9%)	5,908 (61.3%)	1,017,462 (71.0%)
Black	249,026 (17.3%)	2,631 (27.3%)	246,395 (17.2%)
Asian American/Pacific Islander	79,211 (5.5%)	486 (5.0%)	78,725 (5.5%)
American Indian/Alaska Native	18,279 (1.3%)	105 (1.1%)	18,174 (1.3%)
Other	52,019 (3.6%)	379 (3.9%)	51,640 (3.6%)
Unknown	19,624 (1.4%)	108 (1.1%)	19,516 (1.4%)
Missing	1,739 (0.1%)	18 (0.2%)	1,721 (0.1%)
Rank, n (%)			
Junior enlisted	650,885 (45.1%)	2,528 (26.2%)	648,357 (45.2%)
Senior enlisted	526,308 (36.5%)	5,127 (53.2%)	521,181 (36.4%)
Junior officer	181,106 (12.6%)	1,086 (11.3%)	180,020 (12.6%)
Senior officer	69,642 (4.8%)	854 (8.9%)	68,788 (4.8%)
Unknown/missing	15,327 (1.1%)	40 (0.4%)	15,287 (1.1%)
Crude CKD prevalence, n (%)	9,635 (0.7%)	9,635 (100.0%)	0 (0.0%)

Abbreviations: CKD, Chronic kidney disease; FY, fiscal year; IQR, interquartile range; SD, standard deviation.

### CKD Characteristics in Active-Duty Versus non–Active-Duty Individuals

Table 3 summarizes the frequency of ICD-9 code groups in the overall cohort and in active-duty versus non–active-duty subpopulations. The most frequent CKD codes were for not-otherwise-specified CKD, proteinuria, hypertensive kidney disease, and ureteral disease. The active-duty individuals with CKD were more likely to be diagnosed with chronic pyelonephritis, hydronephrosis, ureteral disease, agenesis/cystic disease, CKD of pregnancy, and proteinuria than their non–active-duty counterparts. Non–active-duty individuals with CKD were more likely to be diagnosed with diabetic kidney disease, hypertensive kidney disease, glomerulonephritis, and not otherwise specified. The prevalence of major comorbid conditions in individuals with CKD (hypertension, diabetes, cardiovascular disease, and hyperlipidemia) was each significantly higher in the non–active-duty group than in the active-duty groups.

**Table 3:** Characteristics of CKD in Active-Duty Versus Non–Active-Duty Populations

	FY 2015 CKD	CKD + Active-Duty	CKD + Non–Active-Duty
No. (%)	96,006 (100.0%)	9,635 (10.0%)	86,371 (90.0%)
Age, mean (SD), y	52.9 (17.6)	33.8 (10.0)	55.1 (16.9)
Age, median (IQR), y	55 (41-63)	33 (25-41)	57 (46-64)
Female, n (%)	51,096 (53.2%)	3,021 (31.4%)	48,075 (55.7%)
Race, n (%)			
White	27,659 (28.8%)	5,908 (61.3%)	21,751 (25.2%)
Black	14,356 (15.0%)	2,631 (27.3%)	11,725 (13.6%)
Asian American/Pacific Islander	2,746 (2.9%)	486 (5.0%)	2,260 (2.6%)
American Indian/Alaska Native	436 (0.5%)	105 (1.1%)	331 (0.4%)
Other	1,716 (1.8%)	379 (3.9%)	1,337 (1.6%)
Unknown	13,398 (14.0%)	108 (1.1%)	13,290 (15.4%)
Missing	35,695 (37.2%)	18 (0.2%)	35,677 (41.3%)
Active-duty, n (%)	9,635 (10.0%)	9,635 (100.0%)	0 (0.0%)
Rank, n (%)			
Junior enlisted	7,153 (7.5%)	2,528 (26.2%)	4,625 (5.4%)
Senior enlisted	71,245 (74.2%)	5,127 (53.2%)	66,118 (76.6%)
Junior officer	7,092 (7.4%)	1,086 (11.3%)	6,006 (7.0%)
Senior officer	10,469 (10.9%)	854 (8.9%)	9,615 (11.1%)
Unknown/missing	47 (0.0%)	40 (0.4%)	<11 (0.0%)
ICD-9 diagnosis code frequency, n (%)			
250xx (Diabetic kidney disease)	9,598 (10.0%)	93 (1.0%)	9,505 (11.0%)
403-405xx (Hypertensive kidney disease)	25,825 (26.9%)	689 (7.2%)	25,136 (29.1%)
583xx (Glomerulonephritis)	5,121 (5.3%)	349 (3.6%)	4,772 (5.5%)
585xx (CKD, not otherwise specified)	44,594 (46.5%)	2,001 (20.8%)	42,593 (49.3%)
590xx (Chronic pyelonephritis)	9,190 (9.6%)	1,405 (14.6%)	7,785 (9.0%)
591xx (Hydronephrosis)	8,871 (9.2%)	1,577 (16.4%)	7,294 (8.4%)
593xx (Ureteral Disease)	21,293 (22.2%)	2,867 (29.8%)	18,426 (21.3%)
64xx (CKD in pregnancy)	1,168 (1.2%)	191 (2.0%)	977 (1.1%)
753xx (Agenesis/cystic disease)	6,541 (6.8%)	973 (10.1%)	5,568 (6.5%)
791xx (Proteinuria)	26,272 (27.4%)	2,710 (28.1%)	23,562 (27.3%)
Hypertension, n (%)	57,481 (59.9%)	2,408 (25%)	55,073 (63.8%)
Diabetes, n (%)	31,958 (33.3%)	452 (4.7%)	31,506 (36.5%)
Cardiovascular disease, n (%)	28,613 (29.8%)	931 (9.7%)	27,682 (32.1%)
Hyperlipidemia, n (%)	44,398 (46.3%)	1,564 (16.2%)	42,834 (49.6%)

Note: ICD-9 code frequencies sum to greater than 100% because individuals may have more than 1 diagnosis. P < 0.001 for all comparisons.

Abbreviations: CKD, Chronic kidney disease; FY, fiscal year; ICD-9, International Classification of Diseases, Ninth Revision; IQR, interquartile range; SD, standard deviation.

### Logistic Regression

Table 4 shows the odds ratios for CKD, including the following independent variables: sex, age, race, active-duty versus non–active-duty, and rank. In both unadjusted and adjusted models, an age of greater than 40 years, Black race, and senior enlisted rank group were associated with a higher risk of CKD, whereas active-duty status was associated with a lower risk. Female sex was associated with an increased risk in the unadjusted model but with a decreased risk after adjustment for the other variables. The imputation of missing race moderated the odds ratio for each independent variable; however, they still maintained significance.

**Table 4:** Unadjusted and Adjusted ORs and 95% CIs for CKD in Fiscal Year 2015

Risk Factor	Complete Case Unadjusted OR (95% CI) for CKD	Complete Case Adjusted OR (95% CI) for CKD (n = 2,392,741)	Imputed Race Adjusted OR (95% CI) for CKD (n = 3,344,420)
Female	1.42 (1.40-1.44) *n* = 3,344,419	0.80 (0.79-0.82)	0.94 (0.93-0.95)
Age 40+ y	5.04 (4.97-5.12) *n* = 3,344,420	2.79 (2.72-2.87)	1.63 (1.62-1.64)
Black race vs all others	1.47 (1.45-1.50) *n* = 2,408,123	1.36 (1.34-1.39)	1.16 (1.15-1.17)
Active-duty	0.14 (0.139-0.141) *n* = 3,344,420	0.26 (0.259-0.262)	0.50 (0.49-0.51)
Senior enlisted vs all others	2.38 (2.35-2.42) *n* = 3,328,964	1.33 (1.31-1.36)	1.15 (1.14-1.16)

Abbreviations: CI, Confidence interval; CKD, chronic kidney disease; OR, odds ratio.

## Discussion

This study uses comprehensive claims data to capture CKD prevalence in the adult population served by the MHS. When compared with other large claims databases reported in the US Renal Data System,^5^ the MHS crude CKD prevalence of 2.9% was lower than that reported from the Medicare 5% sample (13.8%) and higher than that from Optum Clinformatics (1.7%). It was comparable with that reported for VA claims data (2.7%); however, CKD prevalence in the VA was much higher (14.9%) when defined by the combination of laboratory data and diagnostic codes instead of just codes alone. It should be emphasized that these are not age-adjusted. With a mean age of 37.6 years, the MHS population was younger than that of VA (62.7 years), Medicare 5% sample (74.7 years), and Optum Clinformatics individuals (44.7 years). The age-adjusted CKD prevalence in the MHS was 4.9% in the FY 2015.

Differences in CKD prevalence across these patient populations are because of various factors, including demographic characteristics (eg, age), the burden of risk factors for CKD, and the method of CKD ascertainment (laboratory-based in National Health and Nutrition Examination Survey vs claim-based in Medicare and Optum Clinformatics vs laboratory and claim-based in VA data). The age-stratified CKD prevalence in the MHS correlated well with reported values in the Medicare 5% sample of 10.1% (for ages 65-74 years), 17.2% (for ages 75-84 years), and 22.6% (for an age greater than 85 years).^5^ Although both the MHS and the VA are models of universal health care access, the MHS demographics are more representative of the US population^5^: more than 75% of the VA population are over the age of 50 years compared with just 25% of the MHS cohort; the MHS population has more women (44.7% vs 10.4%); the MHS provides care to family members, whereas the VA serves veterans only.

The active-duty population is a unique subset of individuals in that they have undergone a screening prior to entry in the military, although neither serum creatinine levels nor imaging are normally obtained. CKD prevalence in the active-duty population was low, with an adjusted odds ratio of 0.5 (95% CI, 0.5-0.5), and the diagnoses observed in this population were more likely to be related to infections or anatomical disorders and less likely to be related to hypertension, diabetes, or glomerulonephritis. CKD in active-duty individuals may thus be less likely to progress to advanced stages or kidney failure.

People of Black race were 20% more likely to have CKD than those of White race. Previous analysis of National Health and Nutrition Examination Survey laboratory data did not find a difference in CKD prevalence in Black versus White individuals but did show a significantly higher rate of progression to kidney failure in Black individuals.^16^ Subsequent studies have shown a higher prevalence of late stage CKD on the basis of estimated glomerular filtration rate among Blacks compared with Whites.^17^^,^^18^ However, the methodology of our study differs from the above studies (code vs laboratory-based diagnosis of CKD).

CKD prevalence was higher in women than in men overall and in the active-duty population but not in the non–active-duty population. In women, the adjusted odds ratio for CKD was 10% lower than that in men, accounting for age, race, active-duty versus non–active-duty status, and rank. Previous studies have reported higher CKD prevalence in women,^19^, ^20^, ^21^ although the rate of progression appears to be slower than that for males.^22^

There are several limitations to this report. First, we cannot make conclusions about causality given the retrospective nature of this study. Second, the diagnosis of CKD was based on ICD-9 coding, which is known to underestimate prevalence compared with more robust measures based on laboratory data^23^^,^^24^ and is subject to misclassification bias. In a recent study of the MHS, Norton et al^25^ showed that the addition of laboratory data to *International Classification of Diseases, Tenth Revision*, codes increased CKD prevalence by a factor of 2.7. Third, there is limited information on kidney failure care because the vast majority of this population is covered by Medicare and treated outside the MHS. Fourth, there were significant missing data on the race variable in the non–active-duty population, although we addressed this limitation using multiple imputations. Although race is not required information for enrollment of non–active-duty individuals in the MHS, the reason why the missing percentage is so high is unknown. Finally, the MHS lacks detailed data on socioeconomic status and ethnicity status.

However, the strength of this study is that it is a large, racially- and geographically-diverse cohort with fewer discrepancies in health care access than those seen in the general US population. The MHS’ patient population, delivery systems, and quality of care parallel those found in private sector health systems in the United States^26^^,^^27^; thus, our findings are likely generalizable to the US patient population at large. The MHS Data Repository is a valuable resource that can be leveraged to further explore quality of care metrics such as the Health People 2030 objectives and to track outcomes such as kidney failure incidence rates, morbidity, and mortality rates in a distinct global health care system.
