# Supplementary material for: Prevalence and Characteristics of CKD in the US Military Health System: A Retrospective Cohort Study
Source: Kidney Med. 2022 May 23;4(7):100487. doi: 10.1016/j.xkme.2022.100487 (PMC9257409; doi:10.1016/j.xkme.2022.100487)
Supplement: Supplementary File (PDF) — Figure S1; Tables S1-S5. [file mmc1.docx]

| **ICD-9 Codes for CKD**  16, 95.4, 189, 189.1, 189.9, 236.91, 250.4, 250.41, 250.42, 250.43, 271.4, 274.1, 283.11, 403, 403.01, 403.1, 403.11, 403.9, 403.91, 404, 404.01, 404.02, 404.03, 404.1, 404.11, 404.12, 404.13, 404.9, 404.91, 404.92, 404.93, 405.01, 440.1, 442.1, 447.3, 453.3, 572.4, 580, 580.4, 580.81, 580.89, 580.9, 581, 581.1, 581.2, 581.3, 581.81, 581.89, 581.9, 582, 582.1, 582.2, 582.4, 582.81, 582.89, 582.9, 583, 583.1, 583.2, 583.4, 583.6, 583.7, 583.81, 583.89, 583.9, 584.5, 584.6, 584.7, 584.8, 584.9, 585.x, 586, 587, 588, 588.1, 588.81, 588.89, 588.9, 589, 590, 590.8, 591, 593.1, 593.3, 593.4, 593.5, 593.6, 593.7, 593.81, 593.89, 593.9, 642.1, 646.2, 753, 753.1, 753.11, 753.12, 753.13, 753.14, 753.15, 753.16, 753.17, 753.19, 753.2, 753.21, 753.22, 753.23, 753.29, 753.3, 753.4, 753.5, 753.6, 753.7, 753.8, 753.9, 791, 791.1, 791.2, 791.3, 791.4, 791.5, 791.6, 791.7, 791.9, 794.4, 585.6, 996.81, V42.0, V45.1. |
| --- |
| **ICD-9 codes for ESKD**  585.6, 996.81, V42.0, V45.1, V56.0, V56.1, V56.2, V56.3, V56.31, V56.32, V56.8, E879.1 |
| **CPT codes for ESKD**  90935, 90937, 90940, 90945, 90947, 90951-90970, 90989, 90993, 90997, 90999 |

**Table S1. International Classification of Diseases, 9th Revision (ICD-9) and Current Procedural Terminology (CPT) codes used to define Chronic Kidney Disease (CKD) and End Stage Kidney Disease (ESKD)**

|  | **FY 2006** | **FY 2007** | **FY 2008** | **FY 2009** | **FY 2010** | **FY 2011** | **FY 2012** | **FY 2013** | **FY 2014** | **FY 2015** |
| --- | --- | --- | --- | --- | --- | --- | --- | --- | --- | --- |
| **No.** | 3,017,699 | 2,831,479 | 2,937,743 | 3,074,443 | 3,184,280 | 3,626,979 | 3,598,402 | 3,568,619 | 3,401,769 | 3,344,420 |
| **Age, mean (SD), years** | 37.3 (15.0) | 38.1 (15.1) | 38.1 (15.1) | 37.9 (15.1) | 37.9 (15.0) | 37.0 (15.1) | 37.2 (15.3) | 37.4 (15.1) | 37.4 (15.4) | 37.6 (15.6) |
| **Age, median (IQR), years** | 34 (24,48) | 36 (25,49) | 36 (25,49) | 35 (25,49) | 35 (25,49) | 33 (24,48) | 34 (24,49) | 34 (24,49) | 34 (24,49) | 34 (24,50) |
| **Female (%)** | 45.9 | 46.8 | 46.5 | 45.9 | 45.4 | 44.9 | 45.1 | 44.9 | 44.8 | 44.7 |
| **Race (%)** |  |  |  |  |  |  |  |  |  |  |
| White | 45.3 | 44.7 | 45.0 | 45.0 | 45.2 | 46.3 | 46.5 | 46.5 | 46.4 | 46.4 |
| Black | 11.5 | 11.4 | 11.5 | 11.7 | 11.7 | 11.8 | 11.9 | 12.2 | 12.5 | 12.7 |
| Asian American/ Pacific Islander | 2.6 | 2.7 | 3.0 | 3.7 | 4.0 | 3.2 | 3.2 | 3.8 | 3.9 | 4.0 |
| American Indian/Alaska Native | 0.8 | 0.8 | 0.8 | 0.9 | 0.9 | 0.8 | 0.8 | 0.8 | 0.8 | 0.8 |
| Other | 2.5 | 2.5 | 2.6 | 2.7 | 2.8 | 3.2 | 2.9 | 2.5 | 2.5 | 2.5 |
| Unknown | 8.3 | 8.4 | 7.8 | 7.3 | 6.7 | 6.0 | 5.8 | 5.7 | 5.6 | 5.7 |
| Missing | 29.0 | 29.4 | 29.2 | 28.8 | 28.7 | 28.7 | 28.9 | 28.6 | 28.3 | 28.0 |
| **Active Duty (%)** | 43.8 | 41.2 | 41.0 | 41.7 | 42.2 | 44.1 | 43.4 | 43.5 | 43.9 | 43.2 |
| **Rank (%)** |  |  |  |  |  |  |  |  |  |  |
| Junior Enlisted | 22.8 | 20.1 | 20.3 | 20.9 | 21.5 | 26.0 | 25.3 | 25.0 | 24.5 | 24.1 |
| Senior Enlisted | 56.5 | 58.5 | 58.5 | 58.1 | 57.6 | 54.0 | 54.4 | 54.6 | 54.7 | 55.0 |
| Junior Officer | 11.0 | 11.1 | 10.9 | 10.7 | 10.7 | 10.4 | 10.6 | 10.6 | 10.9 | 10.9 |
| Senior Officer | 9.3 | 9.9 | 9.9 | 9.9 | 9.7 | 9.1 | 9.3 | 9.4 | 9.5 | 9.6 |
| Unknown/Missing | 0.5 | 0.5 | 0.4 | 0.5 | 0.4 | 0.4 | 0.4 | 0.4 | 0.4 | 0.5 |
| **Crude CKD Prevalence (%)** | 2.1 | 2.5 | 2.6 | 2.8 | 2.9 | 2.8 | 2.8 | 2.7 | 2.8 | 2.9 |
| **ESKD Prevalence (%)** | 0.1 | 0.2 | 0.2 | 0.2 | 0.2 | 0.1 | 0.1 | 0.1 | 0.1 | 0.1 |
| **Age Adjusted CKD Prevalence (%)** | 3.7 | 4.2 | 4.4 | 4.7 | 4.9 | 4.9 | 4.9 | 4.7 | 4.9 | 4.9 |

**Table S2. Demographic characteristics of Military Health System for Fiscal Years (FY) 2006-2015.**

|  | **FY 2006 Total Population** | **Active Duty** | **Non-Active Duty** | **CKD** | **Non-CKD** |
| --- | --- | --- | --- | --- | --- |
| **No. (%)** | 3,017,699 (100) | 1,320,111 (43.8) | 1,697,588 (56.3) | 63,889 (2.1) | 2,953,810 (97.9) |
| **Age, mean (SD), years** | 37.3 (15.0) | 29.0 ( 8.6) | 43.7 (15.8) | 51.0 (18.0) | 37.0 (14.8) |
| **Age , median (IQR), years** | 34 (24,48) | 27 (22,35) | 44 (30,56) | 54 (37,63) | 34 (24,47) |
| **Female (%)** | 1385705 (45.9%) | 218,966 (16.6%) | 1,166,739 (68.7%) | 35,072 (54.9%) | 1,350,633 (45.7%) |
| **Race (%)** |  |  |  |  |  |
| White | 1,368,163 (45.3%) | 960,448 (72.8%) | 407,715 (24.0%) | 15,374 (24.1%) | 1,352,789 (45.8%) |
| Black | 347,147 (11.5%) | 228,445 (17.3%) | 118,702 (7.0%) | 6,250 (9.8%) | 340,897 (11.5%) |
| Asian American/ Pacific Islander | 77,412 (2.6%) | 47,326 (3.6%) | 30,086 (1.8%) | 1,072 (1.7%) | 76,340 (2.6%) |
| American Indian/Alaska Native | 23,679 (0.8%) | 18,631 (1.4%) | 5,048 (0.3%) | 281 (0.4%) | 23,398 (0.8%) |
| Other | 75,652 (2.5%) | 51,452 (3.9%) | 24,200 (1.4%) | 918 (1.4%) | 74,734 (2.5%) |
| Unknown | 251,017 (8.3%) | 12,182 (0.9%) | 238,835 (14.1%) | 14,266 (22.3%) | 236,751 (8.0%) |
| Missing | 874,629 (29.0%) | 1,627 (0.1%) | 873,002 (51.4%) | 25,728 (40.3%) | 848,901 (28.7%) |
| **Active Duty (%)** | 1,320,111 (43.8%) | 1,320,111 (100.0%) | 0 (0.0%) | 7,569 (11.9%) | 1,312,542 (44.4%) |
| **Rank (%)** |  |  |  |  |  |
| Junior Enlisted | 686,423 (22.8%) | 537,765 (40.7%) | 148,658 (8.8%) | 6,362 (10.0%) | 680,061 (23.0%) |
| Senior Enlisted | 1,705,331 (56.5%) | 544,516 (41.3%) | 1,160,815 (68.4%) | 45,590 (71.4%) | 1,659,741 (56.2%) |
| Junior Officer | 331,765 (11.0%) | 161,153 (12.2%) | 170,612 (10.1%) | 4,782 (7.5%) | 326,983 (11.1%) |
| Senior Officer | 280,110 (9.3%) | 63,500 (4.8%) | 216,610 (12.8%) | 7,110 (11.1%) | 273,000 (9.2%) |
| Unknown/Missing | 14,070 (0.5%) | 13,177 (1.0%) | 893 (0.1%) | 45 (0.1%) | 14,025 (0.5%) |
| **Crude CKD Prevalence (%)** | 63,889 (2.1%) | 7,569 (0.6%) | 56,320 (3.3%) | 63,889 (100.0%) | 0 (0.0%) |
| **ESKD Prevalence (%)** | 4,292 (0.1%) | 234 (0.0%) | 4,058 (0.2%) | 4,292 (6.7%) | 0 (0.0%) |
| **Age Adjusted CKD Prevalence (%)** | 3.7 | 0.9 | 4.1 | - | - |

**Table S3A. Characteristics of Active Duty vs. non-Active Duty and Chronic Kidney Disease (CKD) vs. non-CKD in Fiscal Year (FY) 2006.**

|  | **FY 2007 Total Population** | **Active Duty** | **Non-Active Duty** | **CKD** | **Non-CKD** |
| --- | --- | --- | --- | --- | --- |
| **No. (%)** | 2831479 (100) | 1165145 (41.2) | 1666334 (58.9) | 70207 (2.5) | 2761272 (97.5) |
| **Age, mean (SD), years** | 38.1 (15.1) | 29.3 ( 8.5) | 44.2 (15.7) | 51.9 (17.8) | 37.7 (14.9) |
| **Age, median (IQR), years** | 36 (25,49) | 27 (22,35) | 45 (31,56) | 54 (39,63) | 35 (25,48) |
| **Female (%)** | 1,325,069 (46.8%) | 198,094 (17%) | 1,126,975 (67.6%) | 37,809 (53.9%) | 1,287,260 (46.6%) |
| **Race (%)** |  |  |  |  |  |
| White | 1,266,301 (44.7%) | 842,433 (72.3%) | 423,868 (25.4%) | 17,420 (24.8%) | 1,248,881 (45.2%) |
| Black | 323,873 (11.4%) | 200,915 (17.2%) | 122,958 (7.4%) | 7,301 (10.4%) | 316,572 (11.5%) |
| Asian American/ Pacific Islander | 76,983 (2.7%) | 45,487 (3.9%) | 31,496 (1.9%) | 1,292 (1.8%) | 75,691 (2.7%) |
| American Indian/  Alaska Native | 22,861 (0.8%) | 17,600 (1.5%) | 5,261 (0.3%) | 323 (0.5%) | 22,538 (0.8%) |
| Other | 71,607 (2.5%) | 46,552 (4.0%) | 25,055 (1.5%) | 980 (1.4%) | 70,627 (2.6%) |
| Unknown | 237,790 (8.4%) | 10,496 (0.9%) | 227,294 (13.6%) | 15,160 (21.6%) | 222,630 (8.1%) |
| Missing | 832,064 (29.4%) | 1,662 (0.1%) | 830,402 (49.8%) | 27,731 (39.5%) | 804,333 (29.1%) |
| **Active Duty (%)** | 1,165,145 (41.2%) | 1,165,145 (100.0%) | 0 (0.0%) | 7,658 (10.9%) | 1,157,487 (41.9%) |
| **Rank (%)** |  |  |  |  |  |
| Junior Enlisted | 568,113 (20.1%) | 440,470 (37.8%) | 127,643 (7.7%) | 6,081 (8.7%) | 562,032 (20.4%) |
| Senior Enlisted | 1,655,094 (58.5%) | 501,147 (43.0%) | 1,153,947 (69.3%) | 50,640 (72.1%) | 1,604,454 (58.1%) |
| Junior Officer | 313,684 (11.1%) | 148,547 (12.8%) | 165,137 (9.9%) | 5,352 (7.6%) | 308,332 (11.2%) |
| Senior Officer | 280,934 (9.9%) | 61,984 (5.3%) | 218,950 (13.1%) | 8,084 (11.5%) | 272,850 (9.9%) |
| Unknown/Missing | 13,654 (0.5%) | 12,997 (1.1%) | 657 (0.0%) | 50 (0.1%) | 13,604 (0.5%) |
| **CKD Prevalence** | 70,207 (2.5%) | 7,658 (0.7%) | 62,549 (3.8%) | 70,207 (100.0%) | 0 (0.0%) |
| **ESRD Prevalence** | 4,456 (0.2%) | 146 (0.0%) | 4,310 (0.3%) | 4,456 (6.4%) | 0 (0.0%) |
| **Age Adjusted CKD** | 4.2 | 1.4 | 4.6 | - | - |

**Table S3B. Characteristics of Active Duty vs. non-Active Duty and Chronic Kidney Disease (CKD) vs. non-CKD in Fiscal Year (FY) 2007.**

|  | **FY 2008 Total Population** | **Active Duty** | **Non-Active Duty** | **CKD** | **Non-CKD** |
| --- | --- | --- | --- | --- | --- |
| **No. (%)** | 2937743 (100) | 1205546 (41) | 1732197 (59) | 77023 (2.6) | 2860720 (97.4) |
| **Age, mean (SD), years** | 38.1 (15.1) | 29.3 ( 8.5) | 44.2 (15.7) | 52.0 (17.7) | 37.7 (14.8) |
| **Age, median (IQR), years** | 36 (25,49) | 27 (22,35) | 45 (31,56) | 54 (40,63) | 35 (25,48) |
| **Female (%)** | 1,365,329 (46.5%) | 199,332 (16.5%) | 1,165,997 (67.3%) | 41,591 (54.0%) | 1,323,738 (46.3%) |
| **Race (%)** |  |  |  |  |  |
| White | 1,321,438 (45.0%) | 865,389 (71.8%) | 456,049 (26.3%) | 19,997 (26.0%) | 1,301,441 (45.5%) |
| Black | 338,115 (11.5%) | 204,799 (17.0%) | 133,316 (7.7%) | 8,405 (10.9%) | 329,710 (11.5%) |
| Asian American/  Pacific Islander | 88,782 (3.0%) | 53,502 (4.4%) | 35,280 (2.0%) | 1,560 (2.0%) | 87,222 (3.1%) |
| American Indian/  Alaska Native | 24,554 (0.8%) | 18,772 (1.6%) | 5,782 (0.3%) | 350 (0.5%) | 24,204 (0.9%) |
| Other | 77,282 (2.6%) | 50,408 (4.2%) | 26,874 (1.6%) | 1,222 (1.6%) | 76,060 (2.7%) |
| Unknown | 230,317 (7.8%) | 11,212 (0.9%) | 219,105 (12.7%) | 15,300 (19.9%) | 215,017 (7.5%) |
| Missing | 857,255 (29.2%) | 1,464 (0.1%) | 855,791 (49.4%) | 30,189 (39.2%) | 827,066 (28.9%) |
| **Active Duty (%)** | 1,205,546 (41.0%) | 1,205,546 (100.0%) | 0 (0.0%) | 8,214 (10.7%) | 1,197,332 (41.9%) |
| **Rank (%)** |  |  |  |  |  |
| Junior Enlisted | 595,731 (20.3%) | 461,300 (38.3%) | 134,431 (7.8%) | 6,480 (8.4%) | 589,251 (20.6%) |
| Senior Enlisted | 1,717,818 (58.5%) | 516,885 (42.9%) | 1,200,933 (69.3%) | 55,832 (72.5%) | 1,661,986 (58.1%) |
| Junior Officer | 319,472 (10.9%) | 150,802 (12.5%) | 168,670 (9.7%) | 5,702 (7.4%) | 313,770 (11.0%) |
| Senior Officer | 291,638 (9.9%) | 64,048 (5.3%) | 227,590 (13.1%) | 8,950 (11.6%) | 282,688 (9.9%) |
| Unknown/Missing | 13,084 (0.4%) | 12,511 (1.0%) | 573 (0.0%) | 59 (0.1%) | 13,025 (0.5%) |
| **CKD Prevalence** | 77,023 (2.6%) | 8,214 (0.7%) | 68,809 (4.0%) | 77,023 (100.0%) | 0 (0.0%) |
| **ESRD Prevalence** | 4,532 (0.2%) | 159 (0.0%) | 4,373 (0.3%) | 4,532 (5.9%) | 0 (0.0%) |
| **Age Adjusted CKD** | 4.4 | 1.4 | 4.9 | - | - |

**Table S3D. Characteristics of Active Duty vs. non-Active Duty and Chronic Kidney Disease (CKD) vs. non-CKD in Fiscal Year (FY) 2008.**

|  | **FY 2009 Total Population** | **Active Duty** | **Non-Active Duty** | **CKD** | **Non-CKD** |
| --- | --- | --- | --- | --- | --- |
| **No. (%)** | 3074443 (100) | 1280474 (41.6) | 1793969 (58.4) | 85715 (2.8) | 2988728 (97.2) |
| **Age, mean (SD), years** | 37.9 (15.1) | 29.2 ( 8.4) | 44.1 (15.7) | 51.5 (17.6) | 37.5 (14.8) |
| **Age, median (IQR), years** | 35 (25,49) | 27 (22,35) | 45 (31,56) | 54 (39,62) | 35 (24,48) |
| **Female (%)** | 1,411,833 (45.9%) | 208,245 (16.3%) | 1,203,588 (67.1%) | 46,768 (54.6%) | 1,365,065 (45.7%) |
| **Race (%)** |  |  |  |  |  |
| White | 1,382,825 (45.0%) | 903,882 (70.6%) | 478,943 (26.7%) | 22,975 (26.8%) | 1,359,850 (45.5%) |
| Black | 358,086 (11.7%) | 215,422 (16.8%) | 142,664 (8.0%) | 10,219 (11.9%) | 347,867 (11.6%) |
| Asian American/ Pacific Islander | 114,637 (3.7%) | 73,023 (5.7%) | 41,614 (2.3%) | 1,914 (2.2%) | 112,723 (3.8%) |
| American Indian/  Alaska Native | 26,054 (0.9%) | 19,818 (1.6%) | 6,236 (0.4%) | 397 (0.5%) | 25,657 (0.9%) |
| Other | 83,910 (2.7%) | 55,652 (4.4%) | 28,258 (1.6%) | 1,370 (1.6%) | 82,540 (2.8%) |
| Unknown | 223,823 (7.3%) | 10,737 (0.8%) | 213,086 (11.9%) | 15,433 (18.0%) | 208,390 (7.0%) |
| Missing | 885,108 (28.8%) | 1,940 (0.2%) | 883,168 (49.2%) | 33,407 (39.0%) | 851,701 (28.5%) |
| **Active Duty (%)** | 1,280,474 (41.7%) | 1,280,474 (100.0%) | 0 (0.0%) | 9,430 (11.0%) | 1,271,044 (42.5%) |
| **Rank (%)** |  |  |  |  |  |
| Junior Enlisted | 642,102 (20.9%) | 500,174 (39.1%) | 141928 (7.9%) | 7,567 (8.8%) | 634,535 (21.2%) |
| Senior Enlisted | 1,786,904 (58.1%) | 542,800 (42.4%) | 1,244,104 (69.4%) | 62,148 (72.5%) | 1,724,756 (57.7%) |
| Junior Officer | 328,996 (10.7%) | 156,883 (12.3%) | 172,113 (9.6%) | 6,221 (7.3%) | 322,775 (10.8%) |
| Senior Officer | 302,706 (9.9%) | 67,357 (5.3%) | 235,349 (13.1%) | 9,711 (11.3%) | 292,995 (9.8%) |
| Unknown/Missing | 13,735 (0.5%) | 13,260 (1.0%) | 475 (0.0%) | 68 (0.1%) | 13,667 (0.5%) |
| **CKD Prevalence** | 85,715 (2.8%) | 9,430 (0.7%) | 76,285 (4.3%) | 85,715 (100.0%) | 0 (0.0%) |
| **ESRD Prevalence** | 4,578 (0.2%) | 144 (0.0%) | 4,434 (0.3%) | 4,578 (5.3%) | 0 (0.0%) |
| **Age Adjusted CKD** | 4.7 | 1.6 | 5.2 | - | - |

**Table S3D. Characteristics of Active Duty vs. non-Active Duty and Chronic Kidney Disease (CKD) vs. non-CKD in Fiscal Year (FY) 2009.**

|  | **FY 2010 Total Population** | **Active Duty** | **Non-Active Duty** | **CKD** | **Non-CKD** |
| --- | --- | --- | --- | --- | --- |
| **No. (%)** | 3184280 (100) | 1344546 (42.2) | 1839734 (57.8) | 92358 (2.9) | 3091922 (97.1) |
| **Age, mean (SD), years** | 37.9 (15.0) | 29.3 ( 8.4) | 44.1 (15.7) | 51.2 (17.5) | 37.5 (14.8) |
| **Age, median (IQR), years** | 35 (25,49) | 27 (23,35) | 46 (30,56) | 53 (39,62) | 35 (25,48) |
| **Female (%)** | 1,445,825 (45.4%) | 216,317 (16.1%) | 1,229,508 (66.8%) | 50,297 (54.5%) | 1,395,528 (45.1%) |
| **Race (%)** |  |  |  |  |  |
| White | 1,437,738 (45.2%) | 943,576 (70.2%) | 494,162 (26.9%) | 25,512 (27.6%) | 1,412,226 (45.7%) |
| Black | 373,393 (11.7%) | 223,391 (16.6%) | 150,002 (8.2%) | 11,450 (12.4%) | 361,943 (11.7%) |
| Asian American/ Pacific Islander | 127,986 (4.0%) | 81,502 (6.1%) | 46,484 (2.5%) | 2,368 (2.6%) | 125,618 (4.1%) |
| American Indian/  Alaska Native | 27,220 (0.9%) | 20,729 (1.5%) | 6,491 (0.4%) | 436 (0.5%) | 26,784 (0.9%) |
| Other | 89,455 (2.8%) | 60,688 (4.5%) | 28,767 (1.6%) | 1,491 (1.6%) | 87,964 (2.8%) |
| Unknown | 214,304 (6.7%) | 12,458 (0.9%) | 201,846 (11.0%) | 15,002 (16.2%) | 199,302 (6.5%) |
| Missing | 914,184 (28.7%) | 2,202 (0.2%) | 911,982 (49.6%) | 36,099 (39.1%) | 878,085 (28.4%) |
| **Active Duty (%)** | 1,344,546 (42.2%) | 1,344,546 (100.0%) | 0 (0.0%) | 10,647 (11.5%) | 1,333,899 (43.1%) |
| **Rank (%)** |  |  |  |  |  |
| Junior Enlisted | 684,750 (21.5%) | 530,968 (39.5%) | 153,782 (8.4%) | 8,598 (9.3%) | 676,152 (21.9%) |
| Senior Enlisted | 1,834,405 (57.6%) | 563,130 (41.9%) | 1,271,275 (69.1%) | 66,681 (72.2%) | 1,767,724 (57.2%) |
| Junior Officer | 341,169 (10.7%) | 166,303 (12.4%) | 174,866 (9.5%) | 6,925 (7.5%) | 334,244 (10.8%) |
| Senior Officer | 310,286 (9.7%) | 70,645 (5.3%) | 239,641 (13.0%) | 10,101 (10.9%) | 300,185 (9.7%) |
| Unknown/Missing | 13,670 (0.4%) | 13,500 (1.0%) | 170 (0.0%) | 53 (0.1%) | 13,617 (0.4%) |
| **CKD Prevalence** | 92,358 (2.9%) | 10,647 (0.8%) | 81,711 (4.4%) | 92,358 (100.0%) | 0 (0.0%) |
| **ESRD Prevalence** | 4,734 (0.2%) | 154 (0.0%) | 4,580 (0.3%) | 4,734 (5.1%) | 0 (0.0%) |
| **Age Adjusted CKD** | 4.9 | 1.6 | 5.4 | - | - |

**Table S3E. Characteristics of Active Duty vs. non-Active Duty and Chronic Kidney Disease (CKD) vs. non-CKD in Fiscal Year (FY) 2010.**

|  | **FY 2011 Total Population** | **Active Duty** | **Non-Active Duty** | **CKD** | **Non-CKD** |
| --- | --- | --- | --- | --- | --- |
| **No. (%)** | 3626979 (100) | 1600577 (44.1) | 2026402 (55.9) | 99823 (2.8) | 3527156 (97.3) |
| **Age, mean (SD), years** | 37.0 (15.1) | 28.5 ( 8.4) | 43.7 (15.9) | 51.2 (17.7) | 36.6 (14.9) |
| **Age, median (IQR), years** | 33 (24,48) | 26 (22,34) | 45 (29,56) | 54 (38,62) | 33 (24,48) |
| **Female (%)** | 1,630,045 (44.9%) | 261,259 (16.3%) | 1,368,786 (67.6%) | 54,785 (54.9%) | 1,575,260 (44.7%) |
| **Race (%)** |  |  |  |  |  |
| White | 1,678,688 (46.3%) | 1,145,558 (71.6%) | 533,130 (26.3%) | 27,748 (27.8%) | 1,650,940 (46.8%) |
| Black | 426,328 (11.8%) | 263,409 (16.5%) | 162,919 (8.0%) | 12,740 (12.8%) | 413,588 (11.7%) |
| Asian American/ Pacific Islander | 116,422 (3.2%) | 64,749 (4.1%) | 51,673 (2.6%) | 2,411 (2.4%) | 114,011 (3.2%) |
| American Indian/  Alaska Native | 28,723 (0.8%) | 21,614 (1.4%) | 7,109 (0.4%) | 445 (0.5%) | 28,278 (0.8%) |
| Other | 117,675 (3.2%) | 86,545 (5.4%) | 31,130 (1.5%) | 1,701 (1.7%) | 115,974 (3.3%) |
| Unknown | 217,707 (6.0%) | 16,539 (1.0%) | 201,168 (9.9%) | 15,324 (15.4%) | 202,383 (5.7%) |
| Missing | 1,041,436 (28.7%) | 2,163 (0.1%) | 1,039,273 (51.3%) | 39,454 (39.5%) | 1,001,982 (28.4%) |
| **Active Duty (%)** | 1,600,577 (44.1%) | 1,600,577 (100.0%) | 0 (0.0%) | 11,211 (11.2%) | 1,589,366 (45.1%) |
| **Rank (%)** |  |  |  |  |  |
| Junior Enlisted | 941,687 (26.0%) | 736,172 (46.0%) | 205,515 (10.1%) | 10,107 (10.1%) | 931,580 (26.4%) |
| Senior Enlisted | 1,959,606 (54.0%) | 587,649 (36.7%) | 1,371,957 (67.7%) | 71,590 (71.7%) | 1,888,016 (53.5%) |
| Junior Officer | 378,705 (10.4%) | 185,941 (11.6%) | 192,764 (9.5%) | 7,399 (7.4%) | 371,306 (10.5%) |
| Senior Officer | 331,195 (9.1%) | 75,161 (4.7%) | 256,034 (12.6%) | 10,669 (10.7%) | 320,526 (9.1%) |
| Unknown/Missing | 15,786 (0.4%) | 15,651 (1.0%) | 132 (0.0%) | 58 (0.1%) | 15,728 (0.5%) |
| **CKD Prevalence** | 99,823 (2.8%) | 11,211 (0.7%) | 88,612 (4.4%) | 99,823 (100.0%) | 0 (0.0%) |
| **ESRD Prevalence** | 4,696 (0.1%) | 162 (0.0%) | 4,534 (0.2%) | 4,696 (4.7%) | 0 (0.0%) |
| **Age Adjusted CKD** | 4.9 | 1.6 | 5.4 | - | - |

**Table S3F. Characteristics of Active Duty vs. non-Active Duty and Chronic Kidney Disease (CKD) vs. non-CKD in Fiscal Year (FY) 2011.**

|  | **FY 2012 Total Population** | **Active Duty** | **Non-Active Duty** | **CKD** | **Non-CKD** |
| --- | --- | --- | --- | --- | --- |
| **No. (%)** | 3598402 (100) | 1562842 (43.4) | 2035560 (56.6) | 100603 (2.8) | 3497799 (97.2) |
| **Age, mean (SD), years** | 37.2 (15.2) | 28.5 ( 8.4) | 43.9 (15.9) | 51.5 (17.6) | 36.8 (14.9) |
| **Age, median (IQR), years** | 34 (24,49) | 26 (22,34) | 46 (30,56) | 54 (39,62) | 33 (24,48) |
| **Female (%)** | 1,624,035 (45.1%) | 257,667 (16.5%) | 1,366,368 (67.1%) | 55,165 (54.8%) | 1,568,870 (44.9%) |
| **Race (%)** |  |  |  |  |  |
| White | 1,672,358 (46.5%) | 1,127,587 (72.2%) | 544,771 (26.8%) | 28,357 (28.2%) | 1,644,001 (47.0%) |
| Black | 427,648 (11.9%) | 259,597 (16.6%) | 168,051 (8.3%) | 13,282 (13.2%) | 414,366 (11.9%) |
| Asian American/ Pacific Islander | 115,715 (3.2%) | 64,279 (4.1%) | 51,436 (2.5%) | 2,437 (2.4%) | 113,278 (3.2%) |
| American Indian/  Alaska Native | 30,104 (0.8%) | 22,731 (1.5%) | 7,373 (0.4%) | 441 (0.4%) | 29,663 (0.9%) |
| Other | 102,473 (2.9%) | 70,734 (4.5%) | 31,739 (1.6%) | 1,757 (1.8%) | 100,716 (2.9%) |
| Unknown | 209,426 (5.8%) | 16,534 (1.1%) | 192,892 (9.5%) | 14,642 (14.6%) | 194,784 (5.6%) |
| Missing | 1,040,678 (28.9%) | 1,380 (0.1%) | 1,039,298 (51.1%) | 39,687 (39.5%) | 1,000,991 (28.6%) |
| **Active Duty (%)** | 1,562,842 (43.4%) | 1,562,842 (100.0%) | 0 (0.0%) | 11,112 (11.1%) | 1,551,730 (44.4%) |
| **Rank (%)** |  |  |  |  |  |
| Junior Enlisted | 910,888 (25.3%) | 711,633 (45.5%) | 199,255 (9.8%) | 9,673 (9.6%) | 901,215 (25.8%) |
| Senior Enlisted | 1,958,420 (54.4%) | 575,345 (36.8%) | 1,383,075 (68.0%) | 72,687 (72.3%) | 1,885,733 (53.9%) |
| Junior Officer | 379,990 (10.6%) | 186,202 (11.9%) | 193,788 (9.5%) | 7,384 (7.3%) | 372,606 (10.7%) |
| Senior Officer | 334,003 (9.3%) | 74,687 (4.8%) | 259,316 (12.7%) | 10,811 (10.8%) | 323,192 (9.2%) |
| Unknown/Missing | 15,101 (0.4%) | 14,975 (1.0%) | 126 (0.0%) | 48 (0.1%) | 15,053 (0.4%) |
| **CKD Prevalence** | 100,603 (2.8%) | 11,112 (0.7%) | 89,491 (4.4%) | 100,603 (100.0%) | 0 (0.0%) |
| **ESRD Prevalence** | 4,782 (0.1%) | 167 (0.0%) | 4,615 (0.2%) | 4,782 (4.8%) | 0 (0.0%) |
| **Age Adjusted CKD** | 4.9 | 1.8 | 5.4 | - | - |

**Table S3G. Characteristics of Active Duty vs. non-Active Duty and Chronic Kidney Disease (CKD) vs. non-CKD in Fiscal Year (FY) 2012.**

|  | **FY 2013 Total Population** | **Active Duty** | **Non-Active Duty** | **CKD** | **Non-CKD** |
| --- | --- | --- | --- | --- | --- |
| **No. (%)** | 3568619 (100) | 1550394 (43.4) | 2018225 (56.6) | 96771 (2.7) | 3471848 (97.3) |
| **Age, mean (SD), years** | 37.4 (15.3) | 28.4 ( 8.4) | 44.2 (15.9) | 51.8 (17.4) | 36.9 (15.1) |
| **Age, median (IQR), years** | 34 (24,49) | 26 (22,34) | 46 (30,56) | 54 (40,62) | 33 (24,49) |
| **Female (%)** | 1,601,280 (44.9%) | 258,668 (16.7%) | 1,342,612 (66.5%) | 52,827 (54.6%) | 1,548,453 (44.6%) |
| **Race (%)** |  |  |  |  |  |
| White | 1,659,572 (46.5%) | 1,109,693 (71.6%) | 549,879 (27.3%) | 27,789 (28.7%) | 1,631,783 (47.0%) |
| Black | 434,229 (12.2%) | 261,286 (16.9%) | 172,943 (8.6%) | 13,348 (13.8%) | 420,881 (12.1%) |
| Asian American/ Pacific Islander | 135,165 (3.8%) | 82,839 (5.3%) | 52,326 (2.6%) | 2,509 (2.6%) | 132,656 (3.8%) |
| American Indian/  Alaska Native | 28,956 (0.8%) | 21,579 (1.4%) | 7,377 (0.4%) | 425 (0.4%) | 28,531 (0.8%) |
| Other | 87,441 (2.5%) | 55,813 (3.6%) | 31,628 (1.6%) | 1,609 (1.7%) | 85,832 (2.5%) |
| Unknown | 201,854 (5.7%) | 17,592 (1.1%) | 184,262 (9.1%) | 13,366 (13.8%) | 188,488 (5.4%) |
| Missing | 1,021,402 (28.6%) | 1,592 (0.1%) | 1,019,810 (50.5%) | 37,725 (39.0%) | 983,677 (28.3%) |
| **Active Duty (%)** | 1,550,394 (43.5%) | 1,550,394 (100.0%) | 0 (0.0%) | 10,006 (10.3%) | 1,540,388 (44.4%) |
| **Rank (%)** |  |  |  |  |  |
| Junior Enlisted | 892,803 (25.0%) | 709,609 (45.8%) | 183,194 (9.1%) | 8,466 (8.8%) | 884,337 (25.5%) |
| Senior Enlisted | 1,947,038 (54.6%) | 565,807 (36.5%) | 1,381,231 (68.4%) | 70,794 (73.2%) | 1,876,244 (54.0%) |
| Junior Officer | 378,915 (10.6%) | 186,133 (12.0%) | 192,782 (9.6%) | 7,039 (7.3%) | 371,876 (10.7%) |
| Senior Officer | 334,292 (9.4%) | 73,365 (4.7%) | 260,927 (12.9%) | 10,419 (10.8%) | 323,873 (9.3%) |
| Unknown/Missing | 15,571 (0.4%) | 15,480 (1.0%) | 91 (0.0%) | 53 (0.1%) | 15,518 (0.5%) |
| **CKD Prevalence** | 96,771 (2.7%) | 10,006 (0.7%) | 86,765 (4.3%) | 96,771 (100.0%) | 0 (0.0%) |
| **ESRD Prevalence** | 4,720 (0.1%) | 112 (0.0%) | 4,608 (0.2%) | 4,720 (4.9%) | 0 (0.0%) |
| **Age Adjusted CKD** | 4.7 | 1.5 | 5.2 | - | - |

**Table S3H. Characteristics of Active Duty vs. non-Active Duty and Chronic Kidney Disease (CKD) vs. non-CKD in Fiscal Year (FY) 2013.**

|  | **FY 2014 Total Population** | **Active Duty** | **Non-Active Duty** | **CKD** | **Non-CKD** |
| --- | --- | --- | --- | --- | --- |
| **No. (%)** | 3401769 (100) | 1492325 (43.9) | 1909444 (56.1) | 95612 (2.8) | 3306157 (97.2) |
| **Age, mean (SD), years** | 37.4 (15.4) | 28.4 ( 8.4) | 44.4 (16.0) | 52.4 (17.7) | 36.9 (15.1) |
| **Age, median (IQR), years** | 34 (24,49) | 26 (22,34) | 46 (30,56) | 55 (40,63) | 33 (24,49) |
| **Female (%)** | 1,522,339 (44.8%) | 253,702 (17.0%) | 1,268,637 (66.4%) | 51,588 (54.0%) | 1,470,751 (44.5%) |
| **Race (%)** |  |  |  |  |  |
| White | 1,579,127 (46.4%) | 1,061,284 (71.1%) | 517,843 (27.1%) | 27,191 (28.4%) | 1,551,936 (46.9%) |
| Black | 426,391 (12.5%) | 255,665 (17.1%) | 170,726 (8.9%) | 13,708 (14.3%) | 412,683 (12.5%) |
| Asian American/ Pacific Islander | 132,550 (3.9%) | 80,551 (5.4%) | 51,999 (2.7%) | 2,659 (2.8%) | 129,891 (3.9%) |
| American Indian/  Alaska Native | 26,633 (0.8%) | 19,613 (1.3%) | 7,020 (0.4%) | 426 (0.5%) | 26,207 (0.8%) |
| Other | 85,792 (2.5%) | 54,723 (3.7%) | 31,069 (1.6%) | 1,742 (1.8%) | 84,050 (2.5%) |
| Unknown | 190,341 (5.6%) | 18,791 (1.3%) | 171,550 (9.0%) | 13,359 (14.0%) | 176C982 (5.4%) |
| Missing | 960,935 (28.3%) | 1,698 (0.1%) | 959,237 (50.2%) | 36,527 (38.2%) | 924,408 (28.0%) |
| **Active Duty (%)** | 1,492,325 (43.9%) | 1,492,325 (100.0%) | 0 (0.0%) | 9,946 (10.4%) | 1,482,379 (44.8%) |
| **Rank (%)** |  |  |  |  |  |
| Junior Enlisted | 834,326 (24.5%) | 669,699 (44.9%) | 164,627 (8.6%) | 7,771 (8.1%) | 826,555 (25.0%) |
| Senior Enlisted | 1,861,212 (54.7%) | 550,918 (36.9%) | 1,310,294 (68.6%) | 70,328 (73.6%) | 1,790,884 (54.2%) |
| Junior Officer | 369,019 (10.9%) | 184,515 (12.4%) | 184,504 (9.7%) | 7,108 (7.4%) | 361,911 (11.0%) |
| Senior Officer | 322,093 (9.5%) | 72,189 (4.8%) | 249,904 (13.1%) | 10,363 (10.8%) | 311,730 (9.4%) |
| Unknown/Missing | 15,119 (0.4%) | 15,004 (1.0%) | 115 (0.0%) | 42 (0.0%) | 15,077 (0.5%) |
| **CKD Prevalence** | 95,612 (2.8%) | 9,946 (0.7%) | 85,666 (4.5%) | 95,612 (100.0%) | 0 (0.0%) |
| **ESRD Prevalence** | 4,783 (0.1%) | 105 (0.0%) | 4,678 (0.2%) | 4,783 (5.0%) | 0 (0.0%) |
| **Age Adjusted CKD** | 4.9 | 1.7 | 5.4 | - | - |

**Table S3I. Characteristics of Active Duty vs. non-Active Duty and Chronic Kidney Disease (CKD) vs. non-CKD in Fiscal Year (FY) 2014.**

|  | **FY 2006 Total Population** | **Active Duty** | **Non-Active Duty** |
| --- | --- | --- | --- |
| **No. (%)** | 3,017,699 (100) | 1,320,111 (43.8) | 1,697,588 (56.3) |
| **Crude CKD Prevalence (%)** |  |  |  |
| Overall | 2.1 | 0.6 | 3.3 |
| Female | 2.7 | 1.2 | 2.9 |
| Male | 1.8 | 0.5 | 4.5 |
| White | 1.2 | 0.6 | 2.8 |
| Black | 1.9 | 0.9 | 3.9 |
| Asian American/ Pacific Islander | 1.5 | 0.5 | 3.1 |
| American Indian/Alaska Native | 1.3 | 0.7 | 3.4 |
| Other | 1.3 | 0.7 | 2.6 |
| Unknown | 5.6 | 0.3 | 5.9 |
| Missing | 3.1 | - | 3.1 |

**Table S4A. Crude Chronic Kidney Disease (CKD) prevalence by sex and race for Fiscal Year (FY) 2006. Data cells with < 11 individuals are not reported.**

|  | **FY 2007 Total Population** | **Active Duty** | **Non-Active Duty** |
| --- | --- | --- | --- |
| **No. (%)** | 2831479 (100) | 1165145 (41.2) | 1666334 (58.9) |
| **Crude CKD Prevalence (%)** |  |  |  |
| Overall | 2.5 | 0.7 | 3.8 |
| Female | 2.9 | 1.3 | 3.2 |
| Male | 2.2 | 0.6 | 5.1 |
| White | 1.5 | 0.6 | 3.1 |
| Black | 2.4 | 1.1 | 4.6 |
| Asian American/ Pacific Islander | 1.7 | 0.6 | 3.4 |
| American Indian/Alaska Native | 1.4 | 0.7 | 3.9 |
| Other | 1.4 | 0.6 | 2.9 |
| Unknown | 6.2 | 0.4 | 6.5 |
| Missing | 3.4 | - | 3.4 |

**Table S4B. Crude Chronic Kidney Disease (CKD) prevalence by sex and race for Fiscal Year (FY) 2007. Data cells with < 11 individuals are not reported.**

|  | **FY 2008 Total Population** | **Active Duty** | **Non-Active Duty** |
| --- | --- | --- | --- |
| **No. (%)** | 2937743 (100) | 1205546 (41) | 1732197 (59) |
| **Crude CKD Prevalence (%)** |  |  |  |
| Overall | 2.6 | 0.7 | 4.0 |
| Female | 3.1 | 1.3 | 3.4 |
| Male | 2.2 | 0.6 | 5.3 |
| White | 1.6 | 0.7 | 3.4 |
| Black | 2.6 | 1.1 | 4.9 |
| Asian American/ Pacific Islander | 1.8 | 0.6 | 3.6 |
| American Indian/Alaska Native | 1.4 | 0.7 | 3.8 |
| Other | 1.6 | 0.8 | 3.3 |
| Unknown | 6.4 | 0.5 | 6.8 |
| Missing | 3.6 | 0.8 | 3.6 |

**Table S4C. Crude Chronic Kidney Disease (CKD) prevalence by sex and race for Fiscal Year (FY) 2008. Data cells with < 11 individuals are not reported.**

|  | **FY 2009 Total Population** | **Active Duty** | **Non-Active Duty** |
| --- | --- | --- | --- |
| **No. (%)** | 3074443 (100) | 1280474 (41.6) | 1793969 (58.4) |
| **Crude CKD Prevalence (%)** |  |  |  |
| Overall | 2.8 | 0.7 | 4.3 |
| Female | 3.4 | 1.5 | 3.7 |
| Male | 2.4 | 0.7 | 5.5 |
| White | 1.7 | 0.7 | 3.7 |
| Black | 2.9 | 1.2 | 5.5 |
| Asian American/ Pacific Islander | 1.7 | 0.6 | 3.7 |
| American Indian/Alaska Native | 1.6 | 0.7 | 4.2 |
| Other | 1.7 | 0.8 | 3.4 |
| Unknown | 6.7 | 0.4 | 7.0 |
| Missing | 3.8 | 0.9 | 3.8 |

**Table S4D. Crude Chronic Kidney Disease (CKD) prevalence by sex and race for Fiscal Year (FY) 2009. Data cells with < 11 individuals are not reported.**

|  | **FY 2010 Total Population** | **Active Duty** | **Non-Active Duty** |
| --- | --- | --- | --- |
| **No. (%)** | 3184280 (100) | 1344546 (42.2) | 1839734 (57.8) |
| **Crude CKD Prevalence (%)** |  |  |  |
| Overall | 2.9 | 0.8 | 4.4 |
| Female | 3.6 | 1.6 | 3.9 |
| Male | 2.4 | 0.7 | 5.6 |
| White | 1.8 | 0.8 | 3.9 |
| Black | 3.1 | 1.3 | 5.9 |
| Asian American/ Pacific Islander | 1.9 | 0.7 | 4.0 |
| American Indian/Alaska Native | 1.6 | 0.8 | 4.2 |
| Other | 1.7 | 0.8 | 3.6 |
| Unknown | 6.8 | 0.5 | 7.2 |
| Missing | 4.0 | 0.8 | 4.0 |

**Table S4E. Crude Chronic Kidney Disease (CKD) prevalence by sex and race for Fiscal Year (FY) 2010. Data cells with < 11 individuals are not reported.**

|  | **FY 2011 Total Population** | **Active Duty** | **Non-Active Duty** |
| --- | --- | --- | --- |
| **No. (%)** | 3626979 (100) | 1600577 (44.1) | 2026402 (55.9) |
| **Crude CKD Prevalence (%)** |  |  |  |
| Overall | 2.8 | 0.7 | 4.4 |
| Female | 3.4 | 1.3 | 3.7 |
| Male | 2.2 | 0.6 | 5.6 |
| White | 1.7 | 0.6 | 3.9 |
| Black | 3.0 | 1.2 | 5.9 |
| Asian American/ Pacific Islander | 2.1 | 0.6 | 3.9 |
| American Indian/Alaska Native | 1.6 | 0.6 | 4.4 |
| Other | 1.4 | 0.7 | 3.6 |
| Unknown | 6.7 | 0.5 | 7.3 |
| Missing | 3.8 | 0.9 | 3.8 |

**Table S4F. Crude Chronic Kidney Disease (CKD) prevalence by sex and race for Fiscal Year (FY) 2011. Data cells with < 11 individuals are not reported.**

|  | **FY 2012 Total Population** | **Active Duty** | **Non-Active Duty** |
| --- | --- | --- | --- |
| **No. (%)** | 3598402 (100) | 1562842 (43.4) | 2035560 (56.6) |
| **Crude CKD Prevalence (%)** |  |  |  |
| Overall | 2.8 | 0.7 | 4.4 |
| Female | 3.4 | 1.3 | 3.8 |
| Male | 2.3 | 0.6 | 5.5 |
| White | 1.7 | 0.6 | 4.0 |
| Black | 3.1 | 1.2 | 6.1 |
| Asian American/ Pacific Islander | 2.1 | 0.6 | 3.9 |
| American Indian/Alaska Native | 1.4 | 0.6 | 4.0 |
| Other | 1.7 | 0.8 | 3.7 |
| Unknown | 6.7 | 0.5 | 7.2 |
| Missing | 3.8 | 1.2 | 3.8 |

**Table S4G. Crude Chronic Kidney Disease (CKD) prevalence by sex and race for Fiscal Year (FY) 2012. Data cells with < 11 individuals are not reported.**

|  | **FY 2013 Total Population** | **Active Duty** | **Non-Active Duty** |
| --- | --- | --- | --- |
| **No. (%)** | 3568619 (100) | 1550394 (43.4) | 2018225 (56.6) |
| **Crude CKD Prevalence (%)** |  |  |  |
| Overall | 2.7 | 0.7 | 4.3 |
| Female | 3.4 | 1.2 | 3.8 |
| Male | 2.3 | 0.6 | 5.5 |
| White | 1.7 | 0.6 | 4.0 |
| Black | 3.2 | 1.1 | 6.3 |
| Asian American/ Pacific Islander | 1.9 | 0.6 | 3.9 |
| American Indian/Alaska Native | 1.5 | 0.6 | 4.2 |
| Other | 1.9 | 0.7 | 4.0 |
| Unknown | 6.6 | 0.5 | 7.2 |
| Missing | 3.8 | 1.0 | 3.8 |

**Table S4H. Crude Chronic Kidney Disease (CKD) prevalence by sex and race for Fiscal Year (FY) 2013. Data cells with < 11 individuals are not reported.**

|  | **FY 2014 Total Population** | **Active Duty** | **Non-Active Duty** |
| --- | --- | --- | --- |
| **No. (%)** | 3401769 (100) | 1492325 (43.9) | 1909444 (56.1) |
| **Crude CKD Prevalence (%)** |  |  |  |
| Overall | 2.8 | 0.7 | 4.5 |
| Female | 3.4 | 1.2 | 3.8 |
| Male | 2.3 | 0.6 | 5.6 |
| White | 1.7 | 0.6 | 6.0 |
| Black | 3.2 | 1.1 | 6.4 |
| Asian American/ Pacific Islander | 2.0 | 0.6 | 4.1 |
| American Indian/Alaska Native | 1.6 | 0.7 | 4.1 |
| Other | 2.0 | 0.8 | 4.2 |
| Unknown | 6.6 | 0.5 | 7.3 |
| Missing | 3.8 | 0.9 | 3.8 |

**Table S4I. Crude Chronic Kidney Disease (CKD) prevalence by sex and race for Fiscal Year (FY) 2014. Data cells with < 11 individuals are not reported.**

|  | **FY 2015 Total Population** | **Active Duty** | **Non-Active Duty** |
| --- | --- | --- | --- |
| **No. (%)** | 3344420 (100) | 1443268 (43.2) | 1901152 (56.9) |
| **Crude CKD Prevalence (%)** |  |  |  |
| Overall | 2.9 | 0.7 | 4.5 |
| Female | 3.2 | 1.2 | 3.6 |
| Male | 2.2 | 0.5 | 5.2 |
| White | 1.7 | 0.6 | 3.8 |
| Black | 3.0 | 1.0 | 5.9 |
| Asian American/ Pacific Islander | 1.9 | 0.6 | 3.8 |
| American Indian/Alaska Native | 1.6 | 0.6 | 4.2 |
| Other | 1.9 | 0.7 | 3.8 |
| Unknown | 6.3 | 0.5 | 7.0 |
| Missing | 3.5 | 1.0 | 3.5 |

**Table S4J. Crude Chronic Kidney Disease (CKD) prevalence by sex and race for Fiscal Year (FY) 2015. Data cells with < 11 individuals are not reported.**

| FY 2006  Risk Factor | Complete Case  Unadjusted OR (95% CI) for CKD | Complete Case  Adjusted OR (95% CI) for CKD  (n = 2,130,365) | Imputed Race  Adjusted OR (95% CI) for CKD  (n = 3,017,699) |
| --- | --- | --- | --- |
| Female | 1.44 (1.42 – 1.47) n = 3,017,654 | 0.78 (0.76 – 0.80) | 0.93 (0.92 – 0.94) |
| Age 40+ | 3.98 (3.91– 4.05)  n = 3,017,699 | 2.20 (2.13 – 2.26) | 1.50 (1.48 – 1.51) |
| Black race vs. all others | 1.01 (0.99 – 1.04)  n = 2,143,070 | 1.12 (1.09 – 1.15) | 1.02 (1.01 – 1.03) |
| Active Duty | 0.17 (0.169 – 0.171)  n = 3,017,699 | 0.24 (0.23 – 0.25) | 0.50 (0.49 – 0.501) |
| Senior Enlisted vs. all others | 1.93 (1.89 – 1.96) n = 3,003,629 | 1.20 (1.17 – 1.23) | 1.10 (1.09 – 1.11) |

**Table S5A. Odds Ratios (OR) for CKD by female sex, age greater than 40, Black race, active duty status, and senior enlisted rank for Fiscal Year (FY) 2006.**

| FY 2007  Risk Factor | Complete Case  Unadjusted OR (95% CI) for CKD | Complete Case  Adjusted OR (95% CI) for CKD  (n = 1,987,093) | Imputed Race  Adjusted OR (95% CI) for CKD  (n = 2,831,479) |
| --- | --- | --- | --- |
| Female | 1.34 (1.32 – 1.36) n = 2,831,443 | 0.74 (0.72 – 0.76) | 0.92 (0.91 – 0.93) |
| Age 40+ | 4.11 (4.04 – 4.18)  n = 2,831,479 | 2.26 (2.20 – 2.33) | 1.54 (1.52 – 1.55) |
| Black race vs. all others | 1.08 (1.05 – 1.10)  n = 1,999,415 | 1.16 (1.13 – 1.19) | 1.04 (1.03 – 1.06) |
| Active Duty | 0.17 (0.169 – 0.171)  n = 2,831,479 | 0.25 (0.24 – 0.26) | 0.50 (0.50 – 0.51) |
| Senior Enlisted vs. all others | 1.85 (1.82 – 1.88) n = 2,817,825 | 1.20 (1.17 – 1.23) | 1.09 (1.08 – 1.10) |

**Table S5B. Odds Ratios (OR) for CKD by female sex, age greater than 40, Black race, active duty status, and senior enlisted rank for Fiscal Year (FY) 2007.**

| FY 2008  Risk Factor | Complete Case  Unadjusted OR (95% CI) for CKD | Complete Case  Adjusted OR (95% CI) for CKD  (n = 2,067,866) | Imputed Race  Adjusted OR (95% CI) for CKD  (n = 2,937,743) |
| --- | --- | --- | --- |
| Female | 1.36 (1.34 – 1.38) n = 2,937,660 | 0.76 (0.75 – 0.78) | 0.93 (0.92 – 0.94) |
| Age 40+ | 4.19 (4.12 – 4.23)  n = 2,937,743 | 2.37 (2.30 – 2.44) | 1.55 (1.53 – 1.56) |
| Black race vs. all others | 1.13 (1.10 – 1.16)  n = 2,080,488 | 1.18 (1.15 – 1.21) | 1.05 (1.04 – 1.07) |
| Active Duty | 0.17 (0.16 – 0.17)  n = 2,937,743 | 0.25 (0.24 – 0.26) | 0.50 (0.50 – 0.51) |
| Senior Enlisted vs. all others | 1.88 (1.86 – 1.92) n = 2,924,659 | 1.20 (1.17 – 1.23) | 1.10 (1.09 – 1.11) |

**Table S5C. Odds Ratios (OR) for CKD by female sex, age greater than 40, Black race, active duty status, and senior enlisted rank for Fiscal Year (FY) 2008.**

| FY 2009  Risk Factor | Complete Case  Unadjusted OR (95% CI) for CKD | Complete Case  Adjusted OR (95% CI) for CKD  (n = 2,176,021) | Imputed Race  Adjusted OR (95% CI) for CKD  (n = 3,074,443) |
| --- | --- | --- | --- |
| Female | 1.43 (1.41 – 1.45) n = 3,074,370 | 0.81 (0.79 – 0.82) | 0.95 (0.94 – 0.95) |
| Age 40+ | 4.13 (4.06 – 4.19)  n = 3,074,443 | 2.40 (2.33 – 2.46) | 1.53 (1.52 – 1.55) |
| Black race vs. all others | 1.25 (1.22 – 1.28)  n = 2,189,335 | 1.27 (1.25 – 1.30) | 1.10 (1.09 – 1.12) |
| Active Duty | 0.17 (0.16 – 0.17)  n = 3,074,443 | 0.26 (0.25 – 0.27) | 0.51 (0.50 – 0.51) |
| Senior Enlisted vs. all others | 1.92 (1.89 – 1.95) n = 3,060,708 | 1.20 (1.17 – 1.22) | 1.10 (1.09 – 1.11) |

**Table S5D. Odds Ratios (OR) for CKD by female sex, age greater than 40, Black race, active duty status, and senior enlisted rank for Fiscal Year (FY) 2009.**

| FY 2010  Risk Factor | Complete Case  Unadjusted OR (95% CI) for CKD | Complete Case  Adjusted OR (95% CI) for CKD  (n = 2,256,788) | Imputed Race  Adjusted OR (95% CI) for CKD  (n = 3,184,280) |
| --- | --- | --- | --- |
| Female | 1.45 (1.43 – 1.47) n = 3,184,275 | 0.80 (0.79 – 0.82) | 0.95 (0.94 – 0.96) |
| Age 40+ | 4.08 (4.02 – 4.14)  n = 3,184,280 | 2.39 (2.33 – 2.45) | 1.53 (1.52 – 1.54) |
| Black race vs. all others | 1.31 (1.28 – 1.33)  n = 2,270,096 | 1.30 (1.27 – 1.32) | 1.12 (1.11 – 1.13) |
| Active Duty | 0.17 (0.17 – 0.18)  n = 3,184,280 | 0.27 (0.26 – 0.28) | 0.52 (0.51 – 0.52) |
| Senior Enlisted vs. all others | 1.93 (1.90 – 1.96) n = 3,170,610 | 1.22 (1.19 – 1.24) | 1.10 (1.09 – 1.11) |

**Table S5E. Odds Ratios (OR) for CKD by female sex, age greater than 40, Black race, active duty status, and senior enlisted rank for Fiscal Year (FY) 2010.**

| FY 2011  Risk Factor | Complete Case  Unadjusted OR (95% CI) for CKD | Complete Case  Adjusted OR (95% CI) for CKD  (n = 2,569,885) | Imputed Race  Adjusted OR (95% CI) for CKD  (n = 3,626,979) |
| --- | --- | --- | --- |
| Female | 1.51 (1.49 – 1.53) n = 3,626,975 | 0.82 (0.81 – 0.84) | 0.95 (0.94 – 0.96) |
| Age 40+ | 4.40 (4.34 – 4.46)  n = 3,626,979 | 2.52 (2.45 – 2.58) | 1.55 (1.53 – 1.56) |
| Black race vs. all others | 1.37 (1.34 – 1.39)  n = 2,585,543 | 1.32 (1.30 – 1.35) | 1.14 (1.13 – 1.15) |
| Active Duty | 0.15 (0.15 – 0.16)  n = 3,626,979 | 0.26 (0.25 – 0.26) | 0.50 (0.48 – 0.50) |
| Senior Enlisted vs. all others | 2.18 (2.15 – 2.22) n = 3,611,193 | 1.27 (1.25 – 1.29) | 1.11 (1.10 – 1.12) |

**Table S5F. Odds Ratios (OR) for CKD by female sex, age greater than 40, Black race, active duty status, and senior enlisted rank for Fiscal Year (FY) 2011.**

| FY 2012  Risk Factor | Complete Case  Unadjusted OR (95% CI) for CKD | Complete Case  Adjusted OR (95% CI) for CKD  (n = 2,542,717) | Imputed Race  Adjusted OR (95% CI) for CKD  (n = 3,598,402) |
| --- | --- | --- | --- |
| Female | 1.49 (1.47 – 1.51) n = 3,598,400 | 0.82 (0.80 – 0.83) | 0.95 (0.95 – 0.96) |
| Age 40+ | 4.45 (4.38 – 4.51)  n = 3,598,402 | 2.51 (2.45 – 2.58) | 1.56 (1.55 – 1.57) |
| Black race vs. all others | 1.40 (1.37 – 1.43)  n = 2,557,724 | 1.34 (1.31 – 1.37) | 1.15 (1.14 – 1.16) |
| Active Duty | 0.16 (0.15 – 0.16)  n = 3,598,402 | 0.26 (0.25 – 0.27) | 0.51 (0.50 – 0.51) |
| Senior Enlisted vs. all others | 2.21 (2.18 – 2.24) n = 3,583,301 | 1.28 (1.25 – 1.30) | 1.12 (1.11 – 1.13) |

**Table S5G. Odds Ratios (OR) for CKD by female sex, age greater than 40, Black race, active duty status, and senior enlisted rank for Fiscal Year (FY) 2012.**

| FY 2013  Risk Factor | Complete Case  Unadjusted OR (95% CI) for CKD | Complete Case  Adjusted OR (95% CI) for CKD  (n = 2,531,721) | Imputed Race  Adjusted OR (95% CI) for CKD  (n = 3,568,619) |
| --- | --- | --- | --- |
| Female | 1.49 (1.47 – 1.51) n = 3,568,619 | 0.82 (0.80 – 0.83) | 0.95 (0.94 – 0.96) |
| Age 40+ | 4.63 (4.56 – 4.70)  n = 3,568,619 | 2.56 (2.50 – 2.63) | 1.57 (1.55 – 1.58) |
| Black race vs. all others | 1.43 (1.41 – 1.46)  n = 2,547,217 | 1.36 (1.33 – 1.39) | 1.16 (1.15 – 1.18) |
| Active Duty | 0.14 (0.14 – 0.15)  n = 3,568,619 | 0.25 (0.24 – 0.26) | 0.49 (0.49 – 0.50) |
| Senior Enlisted vs. all others | 2.30 (2.27 – 2.33) n = 3,553,048 | 1.30 (1.28 – 1.33) | 1.13 (1.13 – 1.14) |

**Table S5H. Odds Ratios (OR) for CKD by female sex, age greater than 40, Black race, active duty status, and senior enlisted rank for Fiscal Year (FY) 2013.**

| FY 2014  Risk Factor | Complete Case  Unadjusted OR (95% CI) for CKD | Complete Case  Adjusted OR (95% CI) for CKD  (n = 2,425,790) | Imputed Race  Adjusted OR (95% CI) for CKD  (n = 3,401,769) |
| --- | --- | --- | --- |
| Female | 1.46 (1.44 – 1.48) n = 3,401,769 | 0.80 (0.77 – 0.82) | 0.94 (0.94 – 0.95) |
| Age 40+ | 4.84 (4.77 – 4.91)  n = 3,401,769 | 2.69 (2.62 – 2.77) | 1.60 (1.58 – 1.61) |
| Black race vs. all others | 1.44 (1.41 – 1.45)  n = 2,440,834 | 1.34 (1.32 – 1.37) | 1.16 (1.15 – 1.17) |
| Active Duty | 0.14 (0.14 – 0.15)  n = 3,401,769 | 0.25 (0.25 – 0.26) | 0.49 (0.49 – 0.50) |
| Senior Enlisted vs. all others | 2.33 (2.30 – 2.37) n = 3,386,650 | 1.33 (1.30 – 1.36) | 1.14 (1.13 – 1.15) |

**Table S5I. Odds Ratios (OR) for CKD by female sex, age greater than 40, Black race, active duty status, and senior enlisted rank for Fiscal Year (FY) 2014.**


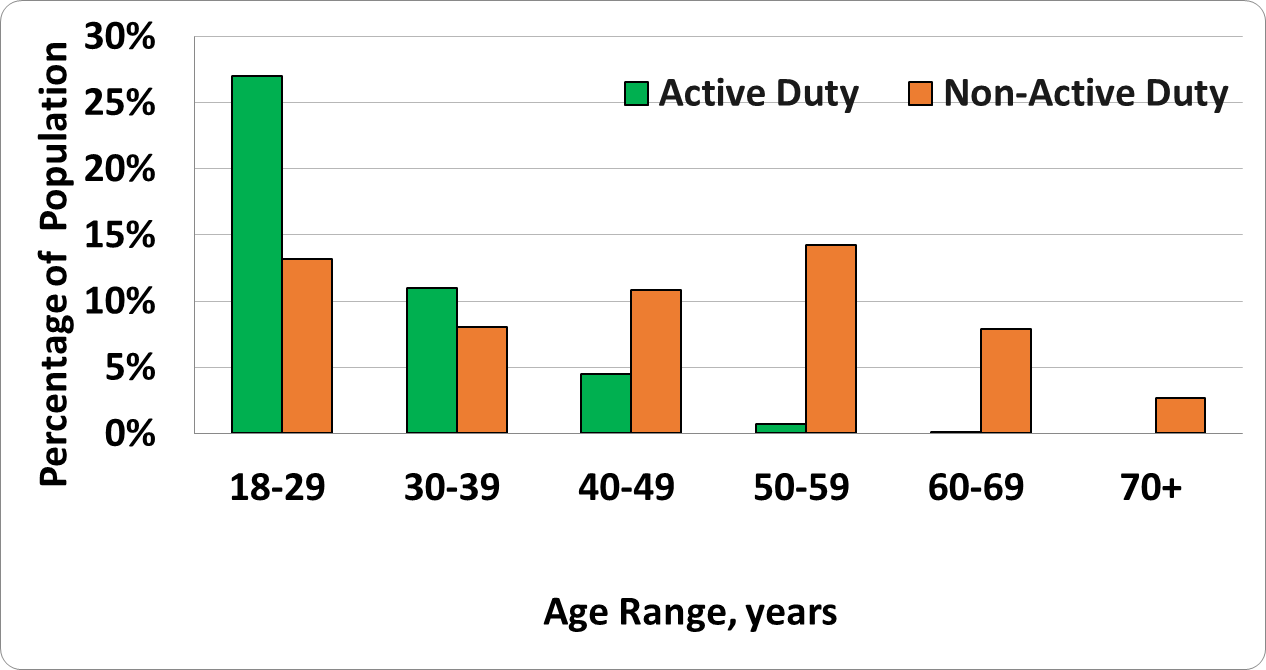


**Figure S1. Age-stratified percentages of Active Duty and non-Active Duty in 2015 cohort.**
